# Evidences of the Low Implication of Mosquitoes in the Transmission of* Mycobacterium ulcerans*, the Causative Agent of Buruli Ulcer

**DOI:** 10.1155/2017/1324310

**Published:** 2017-08-28

**Authors:** Rousseau Djouaka, Francis Zeukeng, Jude Daiga Bigoga, David N'golo Coulibaly, Genevieve Tchigossou, Romaric Akoton, Sylla Aboubacar, Sodjinin Jean-Eudes Tchebe, Clavella Nantcho Nguepdjo, Razack Adeoti, Innocent Djegbe, Manuele Tamo, Wilfred Fon Mbacham, Solange E. Kakou-Ngazoa, Anthony Ablordey

**Affiliations:** ^1^AgroEcoHealth Platform, International Institute of Tropical Agriculture (IITA), 08 P.O. Box 0932, Tri-Postal, Cotonou, Benin; ^2^Faculty of Science, Department of Biochemistry, University of Yaoundé I, P.O. Box 812, Yaoundé, Cameroon; ^3^Department of Technics and Technology, Platform of Molecular Biology, Pasteur Institute Abidjan, P.O. Box 490 Abidjan 01, Abidjan, Côte d'Ivoire; ^4^Faculty of Science and Techniques, University of Abomey-Calavi, P.O. Box 526, Abomey-Calavi, Benin; ^5^Department of Bacteriology, Noguchi Memorial Institute for Medical Research, University of Ghana, P.O. Box 581, Legon, Accra, Ghana

## Abstract

**Background:**

Buruli ulcer (BU) continues to be a serious public health threat in wet tropical regions and the mode of transmission of its etiological agent,* Mycobacterium ulcerans* (*MU*), remains poorly understood. In this study, mosquito species collected in endemic villages in Benin were screened for the presence of* MU*. In addition, the ability of mosquitoes larvae to pick up* MU* from their environment and remain colonized through the larval developmental stages to the adult stage was investigated.

**Methods:**

7,218 adults and larvae mosquitoes were sampled from endemic and nonendemic villages and screened for* MU* DNA targets (*IS2404*,* IS2606*, and KR-B) using qPCR.* Results. MU* was not detected in any of the field collected samples. Additional studies of artificially infected larvae of* Anopheles kisumu* with* MU* strains revealed that mosquitoes larvae are able to ingest and host* MU* during L1, L2, L3, and L4 developmental stages. However, we noticed an absence of these bacteria at both pupae and adult stages, certainly revealing the low ability of infected or colonized mosquitoes to vertically transmit* MU* to their offspring.

**Conclusion:**

The overall findings highlight the low implication of mosquitoes as biological vectors in the transmission cycle of* MU* from the risk environments to humans.

## 1. Introduction

*Buruli ulcer* (BU) is a neglected emerging disease that has recently been reported in some countries as the second most frequent mycobacterial disease in humans after tuberculosis [1–3]. BU continues to be one of the most debilitating cutaneous diseases causing significant morbidity. The disease is characterized by severe subcutaneous necrotic lesions that lead to chronic opened sores and ulcerations, ultimately affecting the bone in extreme cases [4]. Mycolactone, a secreted exotoxin, is the only virulence factor identified to date for* Mycobacterium ulcerans (MU)* [5].

During the last two decades, there has been a reemergence of BU across diverse regions of the world [3, 6, 7]. Its prevalence has increased and currently is seen in over 33 countries worldwide [8]. Although the distribution of BU is global and affects people of all ages, the burden of this disease is most severe in West and Central Africa, as well as some parts of Australia [1, 9, 10]. More than 30,000 cases of BU have been reported in Africa over the last decade and the West African region accounts for more than 67% of the reported cases [6].

The environmental pathogen* Mycobacterium ulcerans (MU)* is the etiologic agent of Buruli ulcer [11]. Merritt et al. [1] provided a series of hierarchical criteria analogous to Koch's postulates and/or the Bradford Hill guidelines emphasizing epidemiological/ecological association and the use of logical inference for establishing cause and effect in biological disease transmission. They further discussed the application of this process to indictment of insect vectors for transmission of* MU*. However, the mode of transmission of* MU* from the risk environments to humans remains unknown and its reservoirs in the environment are still being uncovered [1]. The direct transmission of* MU* from human-to-human is extremely rare and cases usually occur in proximity to slow moving or stagnant bodies of water and among rural and economically deprived populations [12–18]. Recent studies in Australia have demonstrated that mosquitoes may be potential reservoirs or vectors of BU [7, 19–23]. Similarly, a recent study conducted in an endemic area of Cameroon revealed the presence of* MU* molecular markers in hematophagous families of insects like Culicidae (mosquito's family), Ceratopogonidae, and Psychodidae [24]. However, a similar study in an endemic area of Benin did not detect* MU* molecular markers in mosquito species [25]. An experimental laboratory study conducted by Wallace et al. [26] also failed to confirm the implication of mosquitoes as biological vectors in the transmission of BU. These recent studies highlight controversial concerns whether mosquitoes actually play a role in the transmission dynamics of BU. Mosquitoes are the most important group of insects involved in the spread of human and animal diseases [27]. One hypothesis is that they could transmit* MU* to humans. However, there is no scientific or historic precedent for mosquitoes transmitting a bacterium to host in any disease system, either directly or mechanically [1]. In the vector ecology, they may serve as biological vectors and hosts for pathogen replication, or mechanical vectors carrying organisms from hosts to hosts without serving as a site of replication [26, 27]. This last hypothesis has recently been reinforced by Wallace et al. [28] who reported a biologically plausible mechanical transmission mode of BU via natural or anthropogenic skin punctures (trauma). These authors further highlighted that a significant low quantity of* MU* delivered beneath the skin surface of animal (BALB/C mice) by a minor injury created by mosquitoes might cause BU in return [28]. Previously in 1974, Meyers et al. [29] reported that skin trauma could be an important mode of transmitting* MU* infections or of introducing* MU* into the dermis of subcutaneous tissue from superficially contaminated skin. However, Williamson et al. [30] recently established that abrasions (trauma) of the skin in Guinea pig models and subsequent application of* MU* are not sufficient enough to cause an ulcer. Mosquitoes contamination or colonization by* MU* remains an event which has only been reported in Australia and which could vary according to mosquito species. As BU infections occur in humid areas of Africa where high densities of mosquito species are recorded, there is a need to further investigate whether they could be involved in the transmission cycle of BU in African settings and more specifically in Benin.

In this study, we tested the hypothesis of the implication of mosquito species in the transmission of* MU* in an endemic area of Sedje-Denou in the Southern Benin. We further evaluated whether mosquitoes could pick* MU* bacteria from water breeding sites during larval developmental stages leading to colonization and whether colonization continues into the adult stage where they become infective to humans (vertical transmission of* MU* by mosquitoes). Based on these assumptions, we screened wild mosquitoes populations collected from three endemic villages found in Sedje-Denou for molecular targets of* MU*. Coupled to this field based activity, we also investigated the potential for vertical transmission of* MU* within mosquitoes populations using the laboratory strain* Anopheles kisumu*.

## 2. Methods

### 2.1. Ethical Considerations

This research which was mainly laboratory based received administrative clearance from the International Institute of Tropical Agriculture (IITA). In addition, the community consent was obtained prior to mosquitoes sampling in the three villages of Sedje-Denou.

### 2.2. Study Area

**Figure 1:**
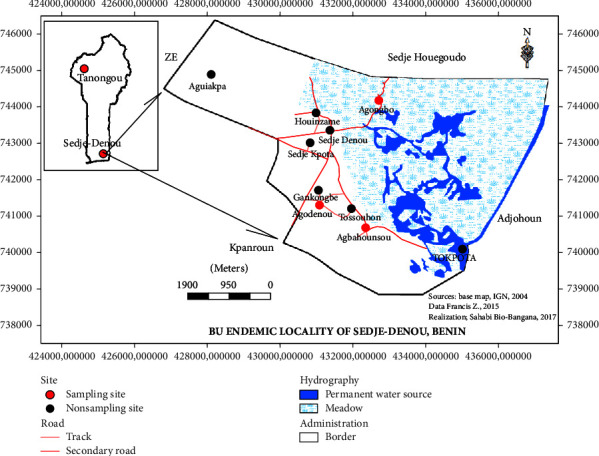
Location map of study sites in the Buruli ulcer endemic area in Southern Benin and the nonendemic area in Northern Benin.

This study was carried out in three endemic communities (Agbahounsou, Agodenou, and Agongbo) of Sedje-Denou (6°32′N and 2°13′E) in the Southern Benin (Figure 1). One nonendemic village, Tanongou (10°48′N and 1°26′E), in the Northern Benin was selected as a negative control village for data comparison. Sedje-Denou (also named Sedje) is located in the Commune of Ze which is the second most endemic locality in Benin with a reported prevalence of 450 cases of BU per 100,000 inhabitants [14]. The presence of rivers and wetlands make this locality an appropriate environment for BU. According to Wagner et al. [31], drainage basins as well as forest land cover with variable wetness patterns are prolific for the growth of MU and associated with higher BU disease prevalence rates. These patterns could also influence the distribution and abundance of vectors, or mediating vector-human interactions. The climate at Sedje is a subequatorial type with two discontinuous dry and wet seasons. The annual average rainfall measures 1,000 mm with an annual average temperature of 24°C and a mean altitude of 20 m. The population of 5,496 inhabitants are distributed into six different villages. Sedje is a rural area where agricultural works being the predominant occupation could contribute to increased exposure to* MU* due to the close spatial proximity with the risk environments [31].

Tanongou is also a rural locality under the Department of Atakora in Northern Benin (Figure 1). This village is administratively subdivided into two close villages named Tanongou 1 and Tanongou 2. BU epidemiological data in Benin show that this locality is a nonendemic area for the disease. The climate is a wet Sudanese type with one long dry season (November to May) and a short rainy season (June to October). This region is dominated by hills of up to 800 m of altitude and several small water bodies, which makes the region colder and relatively wet. Annual rainfall ranges from 1200 to 1300 mm per year, the vegetation is partially of wet savanna type, and the temperature in this part of the country ranges between 23 and 31°C. Agriculture is the prominent activity of the region.

### 2.3. Sampling of Mosquito Species in BU Endemic and Nonendemic Areas

#### 2.3.1. Sampling of Adult Mosquitoes

Field surveys for mosquitoes collections were conducted during rainy seasons at the 3 villages of Sedje-Denou from 2014 to 2016. Similarly, mosquitoes samples were collected at Tanongou (Tanongou 1 + Tanongou 2) during rainy seasons as well. Adult mosquitoes were caught indoors using insecticide spraying technique which is one of the effective methods for collecting indoors resting mosquitoes [32]. Mosquitoes were harvested about twenty minutes after house spraying. They were safely transferred into Petri dishes labeled with room/house references and were taken to the laboratory. In the laboratory, each mosquitoes sample was morphologically identified using Edward identification keys [33]. Mosquitoes were identified to genus and to species. No molecular test was performed for mosquito identification. For each identified species* (Anopheles gambiae s.l., Culex quinquefasciatus, Mansonia africana, and Aedes aegypti)*, pools of 10 mosquitoes each were prepared and kept at −20°C in Eppendorf tubes filled with silica gel. Mosquitoes from Tanongou 1 and Tanongou 2 were pooled and considered as from a single control village of Tanongou.

#### 2.3.2. Sampling of Mosquito Larvae

Mosquito larvae were collected from temporal, semipermanent, and permanent breeding areas using the WHO protocol [34]. Collected larvae were transported to the laboratory where they were morphologically identified and pooled as were the adults. Larvae pools were prepared and stored at −20°C in 70% alcohol.

### 2.4. Molecular Identification of* MU* in Mosquitoes Samples

#### 2.4.1. Extraction of Genomic DNA

Genomic DNA was extracted from a total of 721 pools of mosquitoes samples (adult and larvae) using the phenol/chloroform extraction method described by Sambrook and Russell [35]. Several types of controls were put in place to guide against false positive and negative results. To reduce cross-contaminations, extractions were conducted in batches of 10 pools and the 10 pools completely processed (extraction and PCRs) before moving back to a new set of extractions. Negative controls (nuclease-free water, Sigma-Aldrich) were added at a frequency of 10% (1 control per batch of extraction) to monitor potential cross-contaminations. Pooled mosquitoes samples were ground using an electric grinder in sterile 100 *µ*l 1x PBS and the homogenates were suspended in 300 *µ*l preheated lysis buffer made of 5 M NaCl, 0.5 M EDTA, 1 M Tris-HCl (pH 8.0), 10% SDS, and proteinase K (Qiagen, Hilden). The mixture was heated at 60°C for one hour and DNA extracted with phenol/chloroform/isoamyl acid in the ratio 25 : 24 : 1. This was briefly mixed by a pulse vortex and centrifuged for 2 min at 13,000*g*. The DNA was precipitated by adding 2 volumes of pure ethanol and the mixture was incubated for 2 hours and centrifuged 10 min at 13,000*g*. The DNA was washed by 70% cold ethanol, dried 20 min at room temperature, and eluted in 50 *µ*l of nuclease-free water (Sigma-Aldrich).

#### 2.4.2. Detection of* MU* DNA in Mosquitoes Samples Using TaqMan qPCRs

The TaqMan* IS2404* qPCR analysis described by Fyfe et al. [36] was performed on extracted mosquitoes DNA samples to detect* Mycobacterium* DNA in these samples. A total of 404 pools of DNA samples from adult mosquitoes and a total of 317 pools of DNA samples from mosquitoes larvae from the 4 villages (3 endemic villages and one control village) were subjected to PCR analysis for detecting the presence of* MU* in these wild mosquitoes populations. Briefly, 2.5 *µ*l of the DNA extract was amplified in 12.5 *µ*l PCR mixture using the SensiMix buffer system (BioLine). Each reaction mixture contained 7.5 *µ*l SensiMix (2x SensiMix II probe, No-Rox Mix, BioLine), 0.9 *µ*M* IS2404* primer pair, 0.25 *µ*M* IS2404* probe, a reference Rox dye (Rox Passive Reference Dye, Bio-Rad), and sterile nuclease-free water (Sigma-Aldrich). One positive control (*MU* Agy99 DNA) as well as a no-template negative control (nuclease-free water, Sigma-Aldrich) was used to guide this experiment against false positive and negative results. The amplification process was performed in the Mx3500P automate (MxPro Agilent Technologies, Stratagene Mx3500P) under the following cycling conditions: 50°C for 2 min, 95°C for 10 min, 40 cycles of 95°C for 15 s, and 60°C for 1 min. Negative samples to* IS2404* were diluted 1/10 and resubmitted to molecular analyses for the detection of PCR inhibitors. In addition to the screening of the* IS2404* target, other quantitative real time PCR* IS2606/KR* multiplex assays were performed on* IS2404*-positive samples to screen the presence of* Mycobacterium* conservative insertion sequence* 2606 (IS2606)* and the Ketoreductase B* (KR-B)* domain of the mycolactone polyketide synthase gene of* MU* plasmid (pMUM001) [36]. QPCR mixtures here contained 1 *µ*l of DNA template, 0.9 *µ*M of each primer, 0.25 *µ*M of each probe, 12.5 *µ*l of the SensiMix buffer system (2x SensiMix II probe, No-Rox Mix, BioLine), and nuclease-free water (Sigma-Aldrich) in a total volume of 25 *µ*l. Amplification and detection conditions were performed as described above.

### 2.5. Investigations on the Capability of Mosquitoes to Pick and Host* MU* Bacteria from Larval to Adult Stages (Vertical Transmission of* MU* in Mosquitoes)

This experiment was carried out in the insectary of the AgroEcoHealth Platform of the International Institute of Tropical Agriculture (IITA-Benin). The laboratory strain* Anopheles gambiae kisumu* and* MU* strain isolates were used in this experiment.

#### 2.5.1. The Mosquitoes Strain* Anopheles kisumu*

*Anopheles kisumu* is a reference laboratory strain originating from the Kisumu region in Western Kenya. This strain is commonly used in standardization experiments and is well maintained in most malaria entomology research laboratories.

#### 2.5.2. The Bacterial Strain* Mycobacterium ulcerans* Agy99

*Mycobacterium ulcerans* Agy99 (*MU* Agy99) is a well-characterized Ghanaian human isolate obtained from the Department of Bacteriology at the Noguchi Memorial Institute for Medical Research (NMIMR, Ghana). Agy99 is a reference* MU* strain with a sequenced genome [37].

#### 2.5.3. Experimental Infection of Mosquitoes Larvae with* Mycobacterium ulcerans* and Monitoring of Infected Larvae

Mosquitoes larvae were infected by ingestion of* MU* contaminated Tetramin® Baby Fish Food (Charterhouse Aquatics, London, UK). The infection protocol was adapted from Wallace et al. [26]. Prior to infection, the preserved stock of* MU* strain was diluted in 1X PBS and vortexed 5 min.

*(1) Experimental Infection of Mosquitoes Larvae with MU*. Six groups (4 tests and 2 controls) of 100 eggs of* An. kisumu* each were distributed for rearing into labeled plastic bowls containing 250 ml sterile water. Prior to introducing eggs into bowls, the breeding/rearing water in test groups received 80 mg of Tetramin Baby Fish Food (Charterhouse Aquatics, London, UK) contaminated with 100 *µ*l of* MU* (2.0 10^5^ CFU/ml). The control groups (2 bowls) were prepared in the same way as the test bowls without introducing* MU* contaminated Tetramin Baby Fish Food (Charterhouse Aquatics, London, UK). The mixture (eggs-food-*MU*) was kept in the insectary at 27°C, 75% RH, and 12 : 12 LD for eggs hatching. The first instars larvae progeny (L1) obtained was kept in the contaminated breeding water for ingestion of the bacteria* (MU)* for 24 hours after which the breeding water was completely replaced with a new* MU* free breeding water (water + food only). The L1 larvae were fed with Tetramin and bred till obtaining the second, third, and fourth instars larvae, as well as the pupae and adult mosquitoes. To avoid cross-contaminations during the experiments, all materials and consumables such as rearing bowls, rearing water, and larvae food used for mosquitoes breeding were replaced on daily basis. Rearing waters as well as Tetramin Baby Fish Food were initially tested (qPCR analysis) and confirmed free of* MU* prior to be used in the experiments. Breeding bowls remained covered throughout larval rearing. The entire experiment was repeated thrice to ascertain the accuracy of the data.

*(2) Monitoring of Infected Mosquitoes*. Pools of 10 individuals per developmental stage (egg, L1, L2, L3, L4, pupae, and adult) were prepared from test and control bowls. These pools of individuals were kept in labeled Eppendorf tubes with 70% ethanol and stored at −20°C for molecular screening of* MU*. In addition, we also harvested from breeding water the cuticles from the different larval molting phases and preserved them for similar molecular analysis. Finally, the third group of stored samples was constituted of small volumes (1 ml) of breeding water collected during the entire larval developmental stages. Collected breeding waters were spun at 14,000 rpm for 5 minutes; then, the condensate was vortexed vigorously and 250 *µ*l was used for DNA extraction. The rationale of preserving cuticles and breeding water is to be certain after analysis that the bacterium was effectively ingested by the larvae and is inside the larvae system and not on its skin (due to cuticle colonization). For example, the presence of the bacterium DNA in larvae and its total absence in the water and the cuticle at a given developmental stage will imply that the bacterium was not on the larva skin (colonization of the skin) but is within/inside the larvae. For this infection monitoring experiment, preserved pools of larvae/adults were screened for 2* MU* markers (*IS2404* and KR-B which is more specific to* MU*). A standard curve of the qPCR values (Cts) and the bacterial loads was plotted and this curve was used to determine the bacterial infection rate and to monitor the presence of the bacteria at all larval developmental stages and also at the mosquitoes emergence (the adult stage).

### 2.6. Statistical Analysis

Statistical analysis of generated data was performed using SPSS 17.0 software (SPSS Inc., Chicago IL, USA). Chi-square test was used to set the difference in proportions (mosquitoes distribution and distribution of MU targets between localities and eggs hatching rates). Nonparametric ANOVA test (Kruskal-Wallis) was used to set the difference in means (bacterial loads and “Ct” values according to mosquitoes developmental stage), whereas the Pearson logistic regression test was used to establish the correlation between* MU* bacterial loads and the corresponding “Ct” values (Table 5). A pool of mosquitoes (adults or larvae) was defined infected with* MU* if found positive for the three targets (*IS2404*,* IS2606,* and KR-B) for field screened samples and two targets (*IS2404* and KR) for laboratory infected samples. Two standard curves were plotted from serial dilutions of* MU* strain (Agy99) and the Ct values for* IS2404* and KR-B genes. Based on these standard curves, the cycle threshold (Ct) cut-off was set at less than 35 cycles for* IS2404* and less than 37 cycles for KR-B. Threshold for statistical significance was set at *p* < 5%.

## 3. Results

### 3.1. Distribution of Mosquito' Species Collected in Studied Localities

A total of 4,043 adult mosquitoes were collected during surveyed periods in the three targeted BU endemic villages (Agongbo, Agodenou, and Agbahounsou) and the single BU nonendemic village (Tanongou). 404 pools of 10 adults were generated from sampled mosquitoes which were identified to genus and to species. Pools were grouped by identified species of mosquitoes in each village. Four mosquito species were found in surveyed localities, namely,* Mansonia africana* (34.63%),* Culex quinquefasciatus* (32.95%),* Anopheles gambiae s.l.* (30.05%), and* Aedes aegypti* (2.37%) (Table 1).

In addition to sampled adult mosquitoes, 3,175 mosquitoes larvae were collected from mosquitoes breeding sites found in the endemic villages and the control site. These larvae were used to generate 317 pools of 10 larvae. Larvae identified in the endemic sites included 60.6% of* Culex quinquefasciatus*, 27.86% of* Anopheles gambiae s.l*., and 11.54% of the pupal stage of an* unknown species (unknown* sp.). In the nonendemic control site only two larvae of two genera were detected,* Anopheles gambiae s.l.* (67.02%) and* Culex quinquefasciatus* (32.98%) (Table 1).

### 3.2. Screening of* IS2404*,* IS2606*, and KR-B Targets in Wild Populations of Mosquitoes from Endemic and Nonendemic Localities

#### 3.2.1. Screening of* IS2404*

Out of 301 pools of adult mosquitoes (3,010 mosquitoes) from endemic villages subjected to real time quantitative PCR analysis, 26 pools (8.63%) were found positive to* IS2404* target (Table 2). At Agbahounsou, 8 pools (12.12%) of mosquitoes were found positive to* IS2404*, 12 pools (6.82%) at Agongbo, and 6 pools (10.17%) at Agodenou for this same molecular marker. Unexpectedly, we recorded an identical trend of positive number of pools (10/103, 9.7%) in samples from the nonendemic control site (Table 2).

Out of 223 pools of collected mosquitoes larvae (2,235 mosquitoes larvae) from endemic villages subjected to qPCR analysis, 39 pools (17.49%) were found positive to* IS2404* target with 10 pools (13.51%) at Agbahounsou, 24 pools (32.88%) at Agongbo, and 5 pools (6.58%) at Agodenou. At Tanongou the control site, 11 pools (11.70%) out of 94 tested from 940 mosquitoes larvae were found positive to* IS2404* target (Table 3). No statistical difference was found in the distribution of this target between the test and control localities in both adult and larval mosquitoes (*p* > 0.05).

#### 3.2.2. Screening of* IS2606*

Out of 26 pools of adult mosquitoes tested positive to* IS2404* target in the three endemic villages (Agongbo, Agodenou, and Agbahounsou), none was found to be positive for the* IS2606* target. The same finding was observed after real time quantitative PCR analysis of the 10 pools of mosquitoes tested positive to* IS2404* in the control site. No sample was found positive to* IS2606* in the nonendemic site (Table 2).

However, the* IS2606* target was detected in 3/39 (7.7%) pools of larvae which were positive to* IS2404* target in the endemic sites (Table 3). None of the* IS2404* positive mosquitoes larvae (positive pools) from one endemic site (Agodenou) or the control site were positive for the* IS2606* target (Table 3).

#### 3.2.3. Screening of KR-B

Only one pool (3.84%) out of 26 pools of adult mosquitoes tested positive to* IS2404* target was found positive for the* KR-B* target in samples from the endemic villages. This single KR-B positive pool of mosquitoes belonged to the genus* Anopheles* caught at Agbahounsou. However, it is worth indicating that this unique KR-B (*MU* plasmid marker) positive pool was not found positive to the* IS2606*. None of the 10 pools of* IS2404* positive mosquitoes from the control site tested positive for the* KR-B* target (Table 2).

In addition, none of the mosquitoes larvae that tested positive to* IS2404* target was found to be positive for the* KR-B* in both the endemic and the nonendemic areas (Table 3).

#### 3.2.4. Summary of Results from the Screening of the 3 Targets Related to the Presence of* MU* in Analyzed Wild Populations of Mosquitoes

None of the adult and larvae pools was found to contain the three* MU* targets (*IS2404*,* IS2606,* and* KR-B*). This demonstrated the absence of MU in the wild mosquitoes populations in the endemic region surveyed. Although the* IS2404* target was detected in mosquitoes caught in the nonendemic village, these samples also lacked the three targets related to the presence of* MU* and most likely represent the presence of other environmental mycobacterial species (Tables 2 and 3).

### 3.3. Analysis of the Low Capability of Mosquitoes to Pick and Host* MU* from Larval to Adult Stages

**Figure 2:**
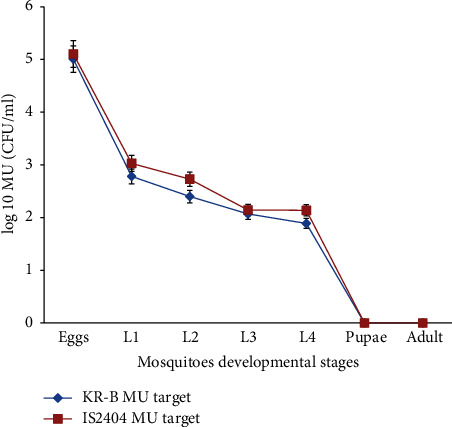
Distribution of average bacterial load during mosquito developmental stages. L1, L2, L3, and L4 correspond to first, second, third, and fourth instars larvae, respectively. Values are given with error bars at 5%.

Following the inoculation of* Anopheles kisumu* eggs in simulated laboratory breeding experiment (bowls containing water, larvae food) fed with* MU*, we recorded an average hatching rate of 94.010 ± 1.289% in the 4 bowls which served as “test bowls” (water + food + MU + eggs of* An. kisumu*) and an average hatching rate of 93.87 ± 0.546% in the 2 bowls serving as “control bowls” (water + food + eggs of* An. kisumu*). Overall, the bacterial load decreased throughout the experiment from the young (1st instars larvae) to the old (pupae and adult stages) developmental stages of* An. kisumu* (Figure 2). No significance difference was observed in the decrease of the bacterial loads throughout the mosquitoes developmental stages in mosquitoes samples (*p* = 0.220), cuticles (*p* = 0.199), and breeding waters (*p* = 0.092).

#### 3.3.1. Distribution of* MU* in First Instars Larvae (L1) of* Anopheles kisumu*

Randomly selected L1 larvae from the 4 “test” bowls (1 pool of 10 L1 larvae per bowl, making a total of 4 pools for the 4 bowls) showed after qPCR analysis that all 4 pools of L1 mosquitoes larvae were infected/colonized by* MU.* Real time PCR analysis targeting the KR-B domain of* MU* revealed a mean Ct value of 31.592 ± 3.151 cycles which corresponds to a mean bacterial load of *E* + 2.779 ± *E* + 0.817 CFU/ml in L1 larvae. The analysis of cuticles (1 pool of 10 cuticles from each bowl, making a total of 4 pools of cuticles from test bowls) released from the metamorphosis of L1 larvae revealed the presence of* MU* in all the 4 pools from “test” bowls (100% infection rate with* MU*, 4/4 pools). The mean Ct value of 36.516 ± 2.096 cycles corresponding to the mean bacterial load of *E* + 1.503 ± *E* + 0.523 CFU/ml was from L1 released cuticles. When the breeding water was analyzed during L1 larval development, the mean planktonic bacterial load in the water was *E* + 3.034 ± *E* + 1.024 CFU/ml, corresponding to a mean Ct value of 30.610 ±2.801 cycles. As observed with larvae and cuticles,* MU* was also detected in all breeding waters (4/4) during the L1 developmental stage of* An. kisumu*.* MU* was not found in L1 larvae, cuticles, or breeding water collected from the 2 bowls constituting the “control group” (Table 4).

#### 3.3.2. Distribution of* MU* in Second Instars Larvae (L2) of* Anopheles kisumu*

Randomly selected L2 larvae pools from the “test” bowls and controls were collected as was the case for the L1 larval stages. All 4 test pools of L2 mosquitoes larvae were infected or colonized by* MU*. Real time PCR of the* MU* KR-B domain of* MU* yielded a mean Ct value of 33.063 ± 2.984 cycles equivalents to a mean bacterial load of *E* + 2.399 ± *E* + 0.773 CFU/ml. Cuticles released from the metamorphosis of L2 larvae had* MU* in 3/4 (75% infection/colonization rate) pools from “test” bowls. The mean Ct value of 36.823 ± 1.652 cycles equivalent to a mean bacterial load of *E* + 1.424 ± *E* + 0.428 CFU/ml was recorded. When the breeding water was analyzed during L2 larval development, the estimated mean planktonic bacterial load found in the water was *E* + 2.705 ± *E* + 0.680 CFU/ml. Bacteria was found in all 4 tests during the L2 developmental stage of* An. kisumu*. Traces of bacteria were not found in L2 larvae, cuticles, or breeding water in the “control group” (Table 4).

#### 3.3.3. Distribution of* MU* in Third Instars Larvae (L3) of* Anopheles kisumu*

Randomly selected L3 larvae pools as previously described for L1 and L2 showed that all 4 pools of L3 mosquitoes larvae were infected or colonized by* MU* and had mean Ct values to the KR-B region of 34.33 ± 3.349 cycles equivalent to a mean bacterial load of *E* + 2.070 ± *E* + 0.031 CFU/ml in L3. The analysis of cuticles only showed the presence of* MU* in 1/4 (25%) pools from “test” bowls. The breeding water during L3 larval development had an estimated mean planktonic bacterial load of *E* + 2.277 ± *E* + 0.023 CFU/ml. MU was not detected in any of the control group samples (Table 4).

#### 3.3.4. Distribution of* MU* in Fourth Instars Larvae (L4) of* Anopheles kisumu*

Randomly selected L4 larvae from the 4 “test” bowls showed that only 3/4 (75%) pools of L4 mosquitoes larvae were infected or colonized by* MU*. The mean Ct value of 35.03 ± 1.177 cycles equivalent to a mean bacterial load of *E* + 1.88 ± *E* + 0.441 CFU/ml was recorded in L4.* MU* was not detected in samples of cuticles released from the metamorphosis of L4 larvae. Breeding water samples had estimated mean planktonic bacterial loads of *E* + 1.652 ± *E* + 0.019 CFU/ml. Three out of 4 (75%) breeding water samples were contaminated with the bacteria during the L4 developmental stage.* MU* was not detected in any of the samples from the L4 control group samples (Table 4).

#### 3.3.5. Distribution of* MU* in Pupae Stages of* Anopheles kisumu*

*MU* was not detected from the randomly selected pupae from the 4 “test” bowls. In addition,* MU* was not detected in the cuticles released from the emergence of adult mosquitoes from pupae. Only one out of 4 (25%) breeding water samples was contaminated with the bacteria* MU* during pupae developmental stage, with a KR-B Ct value of 35.47 cycles. As above,* MU* was not detected in samples constituting the control group (Table 4).

#### 3.3.6. Distribution of* MU* in Adult Stages of* Anopheles kisumu*

Overall,* MU* was not detected in any of adult stage samples or their controls (Table 4).

## 4. Discussion

### 4.1. Wild Populations of Mosquitoes Are Unlikely to Be* MU* Reservoirs in Sedje-Denou

According to WHO, a reservoir is any person, animal, arthropod, plant, soil, or substance, or a combination of these, in which an infectious agent lives and multiplies and where it reproduces itself in such a manner that it can be transmitted to a susceptible host [38]. Difficulties to cultivate* Mycobacterium ulcerans (MU)* from contaminated environmental samples remains the main challenge in the identification of reproductive reservoir(s) for this* Mycobacterium* as well as the understanding of its transmission mode(s) from the* MU* contaminated environment to humans. Most environmental samples that have been identified with* MU* have been classified as “potential reservoirs” [1, 39]. Aquatic water bugs have been shown to replicate* MU* in their salivary glands [40] and* MU* has been successfully recovered by culture from theses insects [11], and thus, the “reservoir” capacity of other “suspected organisms” remains unclear. The aquatic environment has been identified as the most predominant source of* MU* contamination [12–18, 31, 41–45]. This research was conducted in the wet agroecosystem of Sedje-Denou region and more specifically in three endemic villages which served as test sites for this study. From the three thousand and ten adult mosquitoes subjected to real time PCR, twenty-six pools (8.64%) were positive to the insertion sequence* IS2404*, which is not specific enough to infer the presence of* MU*. We recorded the presence of this insertion* (IS2404)* in mosquitoes samples collected from nonendemic location (Tanongou in the Northern Benin). These results further highlight the nonspecificity of this marker for* MU* detection from environmental samples [4, 36, 45]. The use of two additional targets (*IS2606* and KR) to increase the specificity of* MU* detection in our study showed that none of the mosquitoes tested to be simultaneously positive for all three targets. These results certainly confirm the low capability of wild mosquitoes populations to carry* MU* as previously described by others in this same Southern region of Benin [25]. However, our data seems to contradict works conducted in Australia which detected* MU* in mosquitoes samples [7, 19–23, 26]. Johnson et al. [22] described the contamination of mosquito species by* MU* as a consequence of resting and feeding or breeding in storm water drains, whereas Wallace et al. [26], in an experimental study, suggested an unlikely role for mosquitoes as BU biological vectors. In their study using mice and both natural and anthropogenic forms of inoculation, they emphasized that reducing exposure to insect bites and destroying mosquitoes breeding sites around households would break the chain of BU transmission [28]. These series of studies on the role of mosquitoes in the transmission of* MU* show the need of further investigations whether mosquitoes can act as both reservoir and vector of* MU*. In this current study, none of the 2,235 mosquitoes larvae collected from both endemic and nonendemic areas for BU were found to be positive for* MU*, suggesting that mosquitoes larvae in the wild were unlikely to be reservoirs for* MU*. Although our results generated from wild mosquitoes populations are in favor of previous studies conducted in Benin which revealed the inability of mosquitoes to be involved in* MU* transmission [25], a laboratory designed experimental model was designed to better understand the poor implication of mosquitoes in increased number of BU cases in West and Central Africa [1, 6].

### 4.2. Inability of* An. kisumu* Larvae to Pick Up* MU* from Their Environment and Remain Colonized through the Larval Developmental Stages to the Adult Stage

Mosquitoes (Culicidae) development, as characteristic of all holometabolous insects, proceeds through embryonic, larval, pupal, and adult stages that reflect considerable morphological and physiological differences [34]. These stages exhibit distinct niches; larvae and pupae are aquatic while adults are free-flying and terrestrial. In mosquitoes vectors, vertical transmission has been demonstrated for certain pathogens which include yellow fever virus, dengue virus, St. Louis encephalitis virus, Japanese encephalitis virus, and West Nile virus (WNV) [46]. Vertical transmission involves the transmission of pathogens from female mosquitoes to their offspring. The laboratory experimental model showed that mosquitoes larvae readily ingested* MU* and that* MU* colonized the larval stages through the pupal stage. However, at the pupae series of high energy demanding [47], metabolism taking place in the mosquitoes certainly affects* MU* development leading to the clearing of* MU* colonization by the end of pupation and at the adult stage (Figure 2). Our research demonstrated the total absence of* MU* at both pupae and adult stages as reported by Wallace et al. [28] and, thus, highlights the inability of these biting dipterans to act as a good vector/host of* MU* in an endemic environment. Results from this laboratory based experiment are consistent with those obtained from the analysis of thousands of wild populations of mosquitoes collected in the endemic locations which did not show any* MU* colonization through molecular testing. Data published by Wallace et al. [26] suggested that* MU* is unlikely to persist in the mosquito's body system, a behavior which stands as a natural protective mechanism of mosquitoes to bacterial infections. According to Hoxmeier et al. [48], the contamination of* Anopheles gambiae* mosquitoes with* MU* resulted in disruptions to phospholipid metabolic pathways in the mosquitoes, especially the use of glycolipid molecules. Moreover, glycolipids are actively involved in signaling and are mediators in cellular and immune processes [49]. The disruption of synthesis of this molecule probably has a negative impact on the various interactions between* MU* cells and* Anopheles* and the poor capability of mosquitoes to serve as biological vectors for* MU* [45]. Instead of acting as biological vectors for* MU* as described in this study, mosquitoes might act as mechanical vectors as recently described in an experimental study with* Aedes notoscriptus* and BALB/C mice [28]. However, mechanical transmission of* MU* seems to happen only after skin trauma either by an insect bite or by any other environmental stress (e.g., a thorn, penetrating wood splinters, and scorpion stings) [29]. The traumatized skin should initially be colonized by* MU*, a phenomenon that could naturally happen during repetitive contacts with the risk environments such as water bodies or contaminated biofilms [1, 17, 28]. Furthermore, in behavioral study with* Aedes aegypti*, Sanders et al. [50] suggested that if a biofilm of* MU* was on a person, the bacteria may be attracting mosquitoes which in return would lead to a puncture insertion of* MU* as recently reported by Wallace et al. [28]. Although mechanical transmission of* MU* stands as a common mechanism that could correlate transmission studies from both Africa and Australia, Williamson et al. [30] recently established that abrasions (trauma) of the skin in Guinea pig models and subsequent application of* MU* are not sufficient enough to cause an ulcer. Further laboratory and epidemiological studies are therefore required to understand the extent of the mechanical transmission of* MU* and how frequent animals including humans can carry and remain colonized with* MU* on their skin to facilitate such transmission mode.* MU* could be traced from the risk environments to humans or animals directly after they had contact with colonized environments. In such hypothetical situations and for preventive measures, individuals from endemic areas should remain aware and avoid frequent contacts with mosquito's bites by sleeping under mosquitoes bed nets, wearing protective clothing while farming or using clean water for bathing and cleaning [1, 7, 15, 17, 19, 28].

Mosquitoes larvae breeding in an* MU* contaminated water body are capable of ingesting this bacterium as shown by Hoxmeier et al. [48] and Wallace et al. [26] in* Aedes aegypti*,* Aedes albopictus*,* Culex restuans,* and* Ochlerotatus triseriatus* larvae. Although several experimental studies have established the potential of predaceous aquatic insects to temporally maintain* MU* during their developmental stages in water [37, 40], our findings in addition to confirming these previous results also show that* MU* colonization of mosquitoes larvae is very temporal as larvae system is capable of clearing the bacterial load during pupae and adult developmental stages. The vertical transmission of* MU* therefore seems not to be effective in mosquitoes populations as documented with several viruses. The noncontamination/colonization of field-caught mosquito species by* MU* as found in this study might suggest that mosquitoes are unable to move* MU* from one source to another in endemic areas in Benin.

## 5. Conclusion

This study revealed the absence of* MU* in hematophagous mosquitoes trapped in households in BU endemic locations in the Sedje-Denou division in Benin. Using an experimental model, we also showed the inability of laboratory infected or colonized* An. kisumu* larvae to transfer the bacteria to their pupae and the emerging adults. This low ability of mosquitoes to vertically transmit* MU* pathogens to their offspring coupled with the absence of* MU* in field-caught mosquitoes further highlights the low probability of these biting insects as biological vectors for* MU* in endemic villages in Benin. Mosquitoes may therefore not be involved in the dissemination of this pathogen from the risk environments to humans in investigated areas. However, further studies should be performed to evaluate their mechanical implication, before completely excluding whether they are involved or not in the transmission cycle of this emerging disease.

## Figures and Tables

**Table 1:** Distribution of field-caught mosquito species in study sites.

Mosquito species	Developmental stages	Study areas				Total
		Agbahounsou	Agongbo	Agodenou	Tanongou	
*Anopheles gambiae s.l. *	Adult	134	162	119	800	1215
	Larvae	210	110	303	630	1253

*Mansonia africana*	Adult	190	870	200	140	1400

*Culex quinquefasciatus*	Adult	320	690	232	90	1332
	Larvae	450	550	354	310	1664

*Aedes aegypti*	Adult	20	46	25	5	96

*Unknown *sp.	Pupae	80	65	113	0	258

*Total*		*1404*	*2493*	*1346*	*1975*	*7218*

**Table 2:** Distribution of *MU* targets in field-caught adult mosquitoes.

Study sites		Pools of 10 adult mosquitoes analyzed	*IS2404*-qPCR		*KR-*qPCR		*IS2606-qPCR*		MU distribution
			Positive	*P* (%)	Positive	*P* (%)	Positive	*P* (%)	
BU endemic villages	Agbahounsou	66	8	12.12	1	12.5	0	—	Absent
	Agongbo	176	12	6.82	0	—	0	—	Absent
	Agodenou	59	6	10.17	0	—	0	—	Absent

Total		301	26	8.63	1	12.5	0	—	Absent

BU nonendemic village	Tanongou	103	10	9.7	0	—	0	—	Absent

P: percentage of targets distribution. No statistical difference was found in the distribution of *IS2404* target between the endemic and nonendemic localities (*p* = 0.601).

**Table 3:** Distribution of *MU* targets in field collected mosquitoes larvae.

Study sites		Pools of 10 mosquito larvae analyzed	*IS2404*-qPCR		*KR-*qPCR		*IS2606-qPCR*		MU distribution
			Positive	*P* (%)	Positive	*P* (%)	Positive	*P* (%)	
BU endemic villages	Agbahounsou	74	10	13.51	0	—	2	16.67	Absent
	Agongbo	73	24	32.88	0	—	1	5.26	Absent
	Agodenou	76	5	6.58	0	—	0	—	Absent

Total		223	39	17.49	0	—	0	—	Absent

BU nonendemic village	Tanongou	94	11	11.70	0	—	0	—	Absent

P: percentage of targets distribution. No statistical difference was found in the distribution of *IS2404* target between the endemic and nonendemic localities (*p* = 0.347).

**Table 4:** *MU* distribution among mosquitoes developmental stages, cuticles, and breeding waters.

Nature of the samples	Mosquitoes developmental stages	Distribution of *MU* targets		Pool positive/pool tested	Presence of *MU*
		Mean Cts (IS2404)	Mean Cts (KR-B)		
Mosquito^*∗*^	Eggs	19 ± 1.79	21 ± 2.22	4/4	Yes
	L1	27.67 ± 2.66	31.59 ± 3.15	4/4	Yes
	L2	29.92 ± 2.58	33.06 ± 2.98	4/4	Yes
	L3	31.36 ± 2.98	34.33 ± 3.34	4/4	Yes
	L4	31.38 ± 2.20	35.03 ± 1.17	3/4	Yes
	Pupae	NoCt	NoCt	0/4	**No**
	Adults	37.89	NoCt	0/4	**No**

Mosquito cuticles^*∗*^	Eggs	NA	NA	NA	NA
	L1	30.72 ± 1.78	36.51 ± 2.09	4/4	Yes
	L2	34.25 ± 2.83	36.82 ± 1.65	3/4	Yes
	L3	34.13	39.53	1/4	Yes
	L4	NoCt	NoCt	0/4	**No**
	Pupae	38	NoCt	0/4	**No**
	Adults	NA	NA	NA	NA

Mosquito breeding waters^*∗*^	Eggs	18.43 ± 2.03	21.49 ± 1.63	4/4	Yes
	L1	23.04 ± 3.19	30.61 ± 2.80	4/4	Yes
	L2	22.71 ± 2.59	31.88 ± 2.60	4/4	Yes
	L3	28.4 ± 2.86	33.53 ± 3.00	4/4	Yes
	L4	32.00 ± 2.64	35.94 ± 1.04	3/4	Yes
	Pupae	33.65	35.47	1/4	Yes
	Adults	NA	NA	NA	NA

L1, 2, 3, and 4 correspond to first, second, third, and fourth instars larvae, respectively. Yes or no corresponds to the presence or the absence of the bacteria in analyzed samples. NA stands for not applicable. ^*∗*^The bacterial loads did not vary significantly among the developmental stages (*p* < 0.05).

**Table 5:** Characteristics of the standard curves linking “Ct” values of *MU* targets and corresponding bacterial loads (*MU Agy99* serial dilutions).

*MU* targets	Regression coefficient (*R*^2^)	Regression equation (95% CI)	*p* *value*
*IS2404*	0.9955	*Y* = 9.6569 − 0.2396*X*	0.000008
KR-B	0.9968	*Y* = 10.9682 − 0.2592*X*	0.000004

Independent variable, Ct values (cycle threshold); dependent variable, log10 *MU* (CFU/ml).

## References

[1] Merritt R. W., Walker E. D., Small P. L. C. (2010). Ecology and transmission of Buruli ulcer disease: a systematic review. *PLoS Neglected Tropical Diseases*.

[2] Sopoh G. E., Johnson R. C., Chauty A. (2007). Buruli ulcer surveillance, Benin, 2003–2005. *Emerging Infectious Diseases*.

[3] Debacker M., Aguiar J., Steunou C. (2004). *Mycobacterium ulcerans* disease (Buruli ulcer) in rural hospital, southern Benin, 1997–2001. *Emerging Infectious Diseases*.

[4] World Health Organization (2014). *Laboratory Diagnosis of Buruli Ulcer: A Manual for Health Care Providers*.

[5] George K. M., Chatterjee D., Gunawardana G. (1999). Mycolactone: a polyketide toxin from *Mycobacterium ulcerans* required for virulence. *Science*.

[6] World Health Organization (2011). *Buruli ulcer: Number of New Cases of Buruli Ulcer Reported (Per Year)*.

[7] Quek T. Y. J., Athan E., Henry M. J. (2007). Risk factors for *Mycobacterium ulcerans* infection, southeastern Australia. *Emerging Infectious Diseases*.

[8] World Health Organization (2015). *Buruli ulcer (Mycobacterium ulcerans infection)*.

[9] Williamson H. R., Benbow M. E., Campbell L. P. (2012). Detection of *Mycobacterium ulcerans* in the environment predicts prevalence of Buruli ulcer in Benin. *PLoS Neglected Tropical Diseases*.

[10] Francis G., Whitby M., Woods M. (2006). *Mycobacterium ulcerans* infection: a rediscovered focus in the Capricorn Coast region of central Queensland. *Medical Journal of Australia*.

[11] Portaels F., Meyers W. M., Ablordey A. (2008). First cultivation and characterization of *Mycobacterium ulcerans* from the environment. *PLoS Neglected Tropical Diseases*.

[12] Bratschi M. W., Ruf M. T., Andreoli A. (2014). *Mycobacterium ulcerans* persistence at a village water source of Buruli ulcer patients. *PLoS Neglected Tropical Diseases*.

[13] Landier J., Gaudart J., Carolan K. (2014). Spatio-temporal patterns and landscape-associated risk of buruli ulcer in Akonolinga, Cameroon. *PLoS Neglected Tropical Diseases*.

[14] Sopoh G. E., Barogui Y. T., Johnson R. C. (2010). Family relationship, water contact and occurrence of buruli ulcer in Benin. *PLoS Neglected Tropical Diseases*.

[15] Debacker M., Portaels F., Aguiar J. (2006). Risk factors for buruli ulcer, Benin. *Emerging Infectious Diseases*.

[16] Merritt R. W., Benbow M. E., Small P. L. C. (2005). Unraveling an emerging disease associated with disturbed aquatic environments: the case of Buruli ulcer. *Frontiers in Ecology and the Environment*.

[17] Aiga H., Amano T., Cairncross S., Domako J. A., Nanas O.-K., Coleman S. (2004). Assessing water-related risk factors for Buruli ulcer: a case-control study in Ghana. *American Journal of Tropical Medicine and Hygiene*.

[18] Marsollier L., Aubry J., Saint-André J.-P. (2003). Ecology and transmission of *Mycobacterium ulcerans*. *Pathologie Biologie*.

[19] Lavender C. J., Fyfe J. A. M., Azuolas J. (2011). Risk of Buruli ulcer and detection of Mycobacterium ulcerans in mosquitoes in Southeastern Australia. *PLoS Neglected Tropical Diseases*.

[20] Johnson P. D. R., Lavender C. J. (2009). Correlation between buruli Ulcer and vector-borne notifiable diseases, Victoria, Australia. *Emerging Infectious Diseases*.

[21] Quek T. Y. J., Henry J. M., Pasco A. J. (2007). *Mycobacterium ulcerans* infection: factors influencing diagnostic delay. *Medical Journal of Australia*.

[22] Johnson P. D. R., Azuolas J., Lavender C. J. (2007). *Mycobacterium ulcerans* in mosquitoes captured during outbreak of Buruli ulcer, southeastern Australia. *Emerging Infectious Diseases*.

[23] Johnson P. D. R., Stinear T., Small P. L. C. (2005). Buruli ulcer (*M. ulcerans* infection): new insights, new hope for disease control. *PLoS Medicine*.

[24] Le Gall P., Landier J., De Matha Ndengue J. (2015). *Detection of Mycobacterium ulcerans in domestic arthropods in a Buruli ulcer endemic site, Akonolinga, Cameroon*.

[25] Zogo B., Djenontin A., Carolan K. (2015). A field study in Benin to investigate the role of mosquitoes and other flying insects in the ecology of mycobacterium ulcerans. *PLoS Neglected Tropical Diseases*.

[26] Wallace J. R., Gordon M. C., Hartsell L. (2010). Interaction of *Mycobacterium ulcerans* with mosquito species: implications for transmission and trophic relationships. *Applied and Environmental Microbiology*.

[27] Eldridge B. F., Marquardt W. C. (2005). Mosquitoes, the culicidae. *Biology of disease vectors*.

[28] Wallace J. R., Mangas K. M., Porter J. L. (2017). *Mycobacterium ulcerans* low infectious dose and mechanical transmission support insect bites and puncturing injuries in the spread of Buruli ulcer. *PLOS Neglected Tropical Diseases*.

[29] Meyers W. M., Shelly W. M., Connor D. H., Meyers E. K. (1974). Human *Mycobacterium ulcerans* infections developing at sites of trauma to skin. *American Journal of Tropical Medicine and Hygiene*.

[30] Williamson H. R., Mosi L., Donnell R., Aqqad M., Merritt R. W., Small P. L. C. (2014). *Mycobacterium ulcerans* fails to infect through skin abrasions in a guinea pig infection model: implications for transmission. *PLoS Neglected Tropical Diseases*.

[31] Wagner T., Benbow M. E., Brenden T. O., Qi J., Johnson R. C. (2008). Buruli ulcer disease prevalence in Benin, West Africa: associations with land use/cover and the identification of disease clusters. *International Journal of Health Geographics*.

[32] World Health Organization (2006). *Guidelines for Testing Mosquito Adulticides for Indoor Residual Spraying and Treatment of Mosquito Nets*.

[33] Edwards F., Hist B. M. N. (1941). Mosquitoes of the Ethiopian Region III. *Culicine adults and pupae*.

[34] World Health Organization (2003). *Entomologie Du Paludisme Et Contrôle Des Vecteurs, Guide Du Stagiaire*.

[35] Sambrook J., Russell D. W., Sambrook J., Russell D. W. (2001). Purification of nucleic acids by extraction with phenol: chloroform. *Commonly Used Techniques in Molecular Cloning*.

[36] Fyfe J. A. M., Lavender C. J., Johnson P. D. R. (2007). Development and application of two multiplex real-time PCR assays for the detection of *Mycobacterium ulcerans* in clinical and environmental samples. *Applied and Environmental Microbiology*.

[37] Stinear T. P., Seemann T., Pidot S. (2007). Reductive evolution and niche adaptation inferred from the genome of *Mycobacterium ulcerans*, the causative agent of Buruli ulcer. *Genome Research*.

[38] World Health Organization (1998). *Yellow Fever*.

[39] Gryseels S., Amissah D., Durnez L. (2012). Amoebae as potential environmental hosts for *Mycobacterium ulcerans* and other mycobacteria, but doubtful actors in buruli ulcer epidemiology. *PLoS Neglected Tropical Diseases*.

[40] Marsollier L., Robert R., Aubry J. (2002). Aquatic insects as a vector for *Mycobacterium ulcerans*. *Applied and Environmental Microbiology*.

[41] Hayman J. (1991). Postulated epidemiology of *Mycobacterium ulcerans* infection. *International Journal of Epidemiology*.

[42] Kibadi K., Panda M., Tamfum J.-J. M. (2008). New foci of buruli ulcer, Angola and Democratic Republic of Congo. *Emerging Infectious Diseases*.

[43] Duker A. A., Portaels F., Hale M. (2006). Pathways of *Mycobacterium ulcerans* infection: a review. *Environment International*.

[44] Portaels F., Chemlal K., Elsen P. (2001). *Mycobacterium ulcerans* in wild animals. *Revue Scientifique et Technique de l'OIE*.

[45] Morris A. L., Guegan J.-F., Andreou D. (2016). Deforestation-driven food-web collapse linked to emerging tropical infectious disease, *Mycobacterium ulcerans*. *Science Advances*.

[46] Unlu I., MacKay A. J., Roy A., Yates M. M., Foil L. D. (2010). Evidence of vertical transmission of West Nile virus in field-collected mosquitoes. *Journal of Vector Ecology*.

[47] Harker B. W., Behura S. K., Debruyn B. S. (2013). Stage-specific transcription during development of Aedes aegypti. *BMC Developmental Biology*.

[48] Hoxmeier J. C., Thompson B. D., Broeckling C. D. (2015). Analysis of the metabolome of *Anopheles gambiae* mosquito after exposure to *Mycobacterium ulcerans*. *Scientific Reports*.

[49] Atella G. C., Shahabuddin M. (2002). Differential partitioning of maternal fatty acid and phospholipid in neonate mosquito larvae. *Journal of Experimental Biology*.

[50] Sanders M. L., Jordan H. R., Serewis-Pond C. (2017). *Mycobacterium ulcerans* toxin, mycolactone may enhance host-seeking and oviposition behaviour by *Aedes aegypti* (L.) (Diptera: Culicidae). *Environmental Microbiology*.

